# Congenital hypertrophy of the retinal pigment epithelium in Gardner's syndrome

**DOI:** 10.11604/pamj.2014.19.164.4518

**Published:** 2014-10-17

**Authors:** Mina Laghmari, Omar Lezrek

**Affiliations:** 1 University Mohammed V Souissi, Faculty of Medicine and Pharmacy, Rabat, Moroco

**Keywords:** Hypertrophy, retinal pigment epithelium, Gardner′s syndrome

## Image in medicine

**Figure 1:**
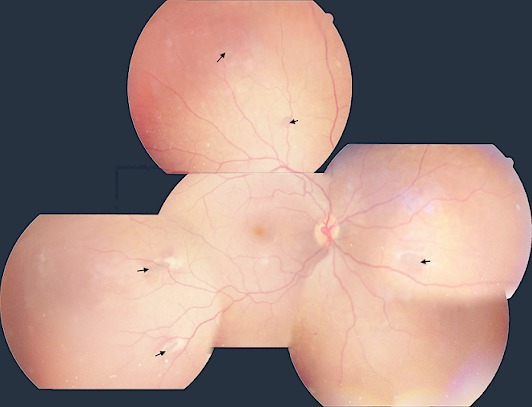
Fundus photomontage: multiple egg-shaped hyperpigmented retinal lesions surrounded by a depigmented halo (black arrows)

A 20-year-old woman presented for a routine eye examination. Her best-corrected visual acuity was 20/20 in the right eye and 20/25 in the left eye. Fundus examination revealed in both eyes the presence of multiple egg-shaped hyperpigmented retinal lesions (at least 4), sur-rounded by a depigmented halo (Figure 1, black arrows). The appearance of these lesions was suggestive of congenital hypertrophy of the retinal pigment epithelium (CHRPE). The patient's medical history and general examination were unremarkable but her family history was pertinent for familial adenomatous polyposis (FAP) in a sister and colon cancer in her mother. The patient underwent colonoscopy and she found to have adenomatous polyps consistent with the diagnosis of Gadner's syndrome (GS). She then was scheduled for bi-yearly screening colonoscopy. CHRPE are congenital hamartomas of retinal pigment epithelium. They can occur as solitary or multiple, they may be found in the normal population and are usually observed during routine ophthalmos copy. Multiple or bilateral CHRPE mayoccur in Familial Adenomatous Polyposis (FAP), an autosomal dominant diseasecaused by mutations in the adenomatous polyposis coli (APC) gene. This entity istermed Gadner's syndrome which include prominent intestinal lesions and extracolonic manifestations such as osteomas, skin tumors, supernumerary teeth, desmoid tumor and CHRPE. Colorectal examination is crucial for early intervention and treatment, as the colon polyps progress to malignancy in nearly 100% of cases. Multiple and bilateral CHRPE in FAP isconsidered a clinical disease marker. However, the absence of CHRPE has no predictive value for absence of GS or FAP.

